# Male Breast Cancer in Serbia: A 33-Year Retrospective Cohort Study of Genetic Predisposition, Clinicopathological Features, and Survival Outcomes

**DOI:** 10.3390/cancers18020326

**Published:** 2026-01-21

**Authors:** Zorka Inić, Milan Žegarac, Ana Krivokuća, Ognjen Živković, Marko Buta, Nikola Vučić, Dobrica Stević, Anđela Milićević, Ivan Marković, Igor Đurišić

**Affiliations:** 1 Clinic of Surgical Oncology, Institute for Oncology and Radiology of Serbia, Pasterova 14, 11000 Belgrade, Serbia; milan_zegarac@yahoo.com (M.Ž.); markobuta@gmail.com (M.B.); nikola.vucic91@gmail.com (N.V.); d.stevic93@live.com (D.S.); ivanmarkovic66@yahoo.com (I.M.); drigordjurisic@gmail.com (I.Đ.); 2 Faculty of Medicine, University of Belgrade, Dr Subotića 8, 11000 Belgrade, Serbia; 3 Department for Genetic Counseling, Institute for Oncology and Radiology of Serbia, Pasterova 14, 11000 Belgrade, Serbia; krivokuca.ana@gmail.com; 4 Department of Pathology, Institute for Oncology and Radiology of Serbia, Pasterova 14, 11000 Belgrade, Serbia; drognjenzivkovic@yahoo.com (O.Ž.); andjela1204@hotmail.com (A.M.)

**Keywords:** male breast cancer, genetic testing, clinicopathological characteristics

## Abstract

**Background/Objectives**: Male breast cancer (MBC) is rare, accounting for less than 1% of all breast cancers. Given its low incidence, male breast cancer (MBC) remains understudied; this 33-year Serbian cohort was assessed for clinicopathological features, therapeutic approaches, genetic alterations, and survival. **Methods**: We retrospectively analyzed MBC patients diagnosed between 1991 and 2024 at the Institute for Oncology and Radiology of Serbia. Data included demographics, tumor characteristics, and stage, treatment, hormone receptor and HER2 status, Ki-67 index, genetic testing, and survival. **Results**: A total of 191 patients were identified (median age 66). Family history was negative in 91% and positive in 5.8%. T2 tumors were most frequent (36%), and 96% presented without metastasis. Mastectomy with axillary or sentinel lymph node dissection was performed in 78.5%. Neoadjuvant chemotherapy and radiotherapy were administered in 5.8% and 8.4%. Estrogen receptor positivity was 72%, progesterone receptor 88%, HER2 overexpression 11.0%, and triple-negative tumors 2.6% (40% with axillary involvement). High Ki-67 (≥15%) was recorded in 28.8%. Adjuvant chemotherapy, radiotherapy, and hormone therapy were given in 36%, 58%, and 68%. Among 37 genetically tested patients, seven had pathogenic variants (BRCA1, BRCA2, CHEK2, PALB2). Disease recurrence occurred in 30%. Median follow-up was 53 months. Median disease-free survival (DFS) was 82 months (1-, 2-, 5-, 10-year DFS: 87%, 73%, 57%, 39%). Median overall survival (OS) 131 months (1-, 2-, 5-, 10-year OS: 95%, 93%, 73%, 53%). **Conclusions**: This long-term cohort highlights the predominance of hormone-receptor positivity, the infrequency of germline mutations, and moderate survival rates, informing patient management and guiding future studies.

## Simple Summary

Male breast cancer is a very rare type of malignancy, and little is known about its causes, characteristics, and long-term outcomes. This study analyzed 191 patients diagnosed with breast cancer in Serbia over 33 years to better understand the features of the disease, treatment approaches, genetic risk factors, and survival outcomes. We found that most patients had hormone-sensitive tumors, only a few carried inherited genetic mutations, and survival rates were moderate over the long term. By providing detailed information on the clinical and genetic aspects of male breast cancer, this research can help doctors make more informed decisions about diagnosis and treatment. It also provides valuable data for future studies, helping the research community improve the understanding and management of this uncommon disease.

## 1. Introduction

Male breast cancer (MBC) is an uncommon condition, accounting for less than 1% of all breast cancer cases and approximately 0.5–1% of all male malignancies [1,2,3]. Due to its low incidence, the development of large prospective trials has been limited, so most diagnostic and therapeutic approaches are extrapolated from female breast cancer [3,4,5].

Comprehensive population-based analyses conducted at the global, regional, and national levels, in conjunction with epidemiological studies, provide robust evidence of a gradual yet sustained increase in the incidence of male breast cancer over recent decades, predominantly affecting older age groups [6,7,8]. Several risk factors have been implicated, including aging, hormonal imbalances, obesity, liver disease, prior chest irradiation, and hereditary susceptibility, particularly BRCA2 mutations, which confer a markedly increased lifetime risk compared to the general male population [5,9,10,11].

Molecular profiling has demonstrated that, similar to female breast cancer, MBC can be classified into four major subtypes—luminal A-like, luminal B-like, HER2-positive, and triple-negative—based on estrogen receptor (ER), progesterone receptor (PR), HER2 status, and proliferation indices such as Ki-67 [9,12,13]. Available data suggest that luminal subtypes predominate in men, while triple-negative tumors are relatively uncommon compared with women [2,12,13]. Recent genomic and transcriptomic profiling studies indicate that male breast cancer (MBC) harbors distinct molecular features and mutation patterns compared with female breast cancer, with significant differences in the frequency of key somatic alterations and gene expression signatures across subtypes [14,15].

Recent guidelines from the American Society of Clinical Oncology (ASCO) and other expert groups recommend endocrine therapy, typically tamoxifen, as the cornerstone of adjuvant hormone treatment for receptor-positive MBC; however, the long-term risk of late recurrence and breast-cancer-specific mortality remains substantial [3,4]. Additionally, quality-of-life data suggest that systemic therapies may have a significant impact on long-term well-being [10].

This study aimed to provide a comprehensive single-center analysis of 191 male breast cancer patients, focusing on demographic and clinicopathological characteristics, molecular subtype distribution, treatment modalities, genetic findings, and disease-free survival (DFS) and overall survival (OS). To our knowledge, this represents one of the largest institutional cohorts of MBC in the region, with detailed pathological and long-term survival data, contributing valuable insights for clinical management and future research.

## 2. Materials and Methods

### 2.1. Study Design and Population

This retrospective cohort study included male patients diagnosed with histologically confirmed breast carcinoma between January 1991 and December 2024 at the Institute for Oncology and Radiology of Serbia, a national tertiary referral center. A total of 191 consecutively treated patients with available clinical, pathological, treatment-related, genetic, and follow-up data were identified through institutional medical records and electronically archived pathology databases.

Exclusion criteria included patients with non-epithelial breast malignancies, metastatic disease to the breast from non-breast primary tumors, female patients, and cases with insufficient documentation precluding confirmation of diagnosis or follow-up.

Due to the retrospective design and long study period, some variables were incomplete, particularly biomarker data such as Ki-67 and HER2 status in earlier decades. Missing or incomplete data were not imputed. Instead, analyses were conducted using an available-case approach, and tumors lacking sufficient biomarker information were categorized as luminal not otherwise specified (luminal NOS), as described below.

### 2.2. Data Collection and Variables

Clinical, histopathological, therapeutic, and survival data were systematically extracted from existing databases. The following variables were collected:

- Demographics: age at diagnosis, age category (≥50 years), family history of breast cancer.
- Disease characteristics: TNM stage, clinical stage, pathological tumor size (pT).
- Pathology: histologic grade, axillary lymph node status (number of removed nodes, number of positive nodes).
- Biomarkers: ER, PR, HER2 status, Ki-67 index.
- Molecular subtype: luminal A-like, luminal B-like, HER2-positive, triple-negative, or luminal not otherwise specified (NOS), assigned according to contemporary immunohistochemical surrogate definitions.
- Germline BRCA testing was not systematically performed in all patients. For statistical analysis, patients without available genetic testing results were assigned to a predefined category (BRCA = 4), indicating presumed BRCA wild-type status.
- Treatment: data were collected on systemic therapies, including chemotherapy, endocrine therapy, radiotherapy, and targeted therapies where applicable and available, including HER2-directed agents. Additionally, information on surgical procedures, adjuvant chemotherapy, radiotherapy, hormonal therapy, other systemic treatments, and palliative radiotherapy was recorded.
- Outcomes: relapse, disease-free survival (DFS), overall survival (OS), vital status, results of BRCA and multigene panel testing where available.

### 2.3. Definitions

- Disease-free survival (DFS) was defined as the interval from primary surgery to first documented locoregional or distant recurrence, or death from any cause, whichever occurred first.
- Overall survival (OS) was defined as the interval from diagnosis to death from any cause or last follow-up.
- Molecular subtype classification: molecular subtypes were assigned using immunohistochemical surrogate definitions based on estrogen receptor (ER), progesterone receptor (PR), HER2 status, Ki-67 proliferation index, and histologic grade, as commonly applied in the literature for breast cancer subtype approximation.

As intrinsic molecular subtyping was not performed, the classification reflects surrogate, receptor-based categorization rather than a formally validated molecular taxonomy.

- Tumors were classified as luminal A-like if they were ER- and/or PR-positive, HER2-negative, with a low proliferation index (Ki-67 < 15%) and low to intermediate histologic grade.
- Luminal B-like tumors were defined as ER- and/or PR-positive cancers with either HER2 overexpression and/or a high proliferation index (Ki-67 ≥ 15%), regardless of grade.
- Tumors that were hormone-receptor-positive but lacked sufficient data on Ki-67 and/or HER2 status to allow reliable subclassification were categorized as luminal not otherwise specified (luminal NOS).
- HER2-positive tumors were defined by HER2 overexpression irrespective of hormone receptor status, and triple-negative tumors were defined by the absence of ER, PR, and HER2 expression.
- Hormone receptor positivity (ER and/or PR) was assessed as an individual biomarker characteristic, whereas luminal subtype assignment required the simultaneous availability of ER/PR status, HER2 status, and Ki-67 index. Consequently, not all hormone-receptor-positive tumors could be included in luminal subtype categories.
- Terminology clarification: the term “not otherwise specified” (NOS) is used in this manuscript in two distinct and non-overlapping contexts.
- Invasive ductal carcinoma, NOS refers to a histopathological diagnosis according to the World Health Organization (WHO) classification and denotes tumors that do not fulfill criteria for a specific histologic special type.
- In contrast, luminal NOS represents an immunohistochemical, receptor-based category applied to hormone-receptor-positive tumors that could not be further subclassified into luminal A-like or luminal B-like subgroups due to missing or incomplete data on Ki-67 and/or HER2 status.
- These two uses of the term NOS refer to different classification levels—histologic versus molecular surrogate—and should not be considered interchangeable.
- Ki-67 assessment and handling of missing data: The Ki-67 proliferation index was assessed by immunohistochemistry and reported as the percentage of positively stained tumor cell nuclei. A cutoff value of 15% was used to distinguish low from high proliferative activity, consistent with commonly used immunohistochemical surrogate definitions of luminal breast cancer reported in the literature [13].

Due to the retrospective nature of the study and the long study period, Ki-67 data were not available for all cases, particularly for tumors diagnosed in earlier years. Hormone-receptor-positive tumors with missing Ki-67 and/or incomplete HER2 information were therefore not assigned to luminal A-like or luminal B-like subgroups and were classified as luminal not otherwise specified (luminal NOS).

### 2.4. Genetic Testing and BRCA Category Definitions

Genetic testing approaches varied over the study period and included either BRCA1/2-only analysis or multigene panel testing, depending on clinical indication and testing availability at the time of diagnosis.

To harmonize heterogeneous genetic results, patients were classified into predefined BRCA status categories (BRCA = 0–6), encompassing pathogenic variants in BRCA1 (BRCA = 1) or BRCA2 (BRCA = 2), or pathogenic variants in other breast cancer predisposition genes (CHEK2, BRCA = 5, and PALB2, BRCA = 6), or the absence of pathogenic BRCA variants after testing (BRCA = 0). Patients without available germline genetic testing were assigned to a predefined category (BRCA = 4), representing a presumed BRCA-wild-type group for analytical purposes.

### 2.5. Statistical Analysis

Descriptive statistics were used to summarize patient characteristics. Continuous variables were presented as mean, standard deviation (SD), median, and range; categorical variables as counts and percentages. DFS and OS were estimated using the Kaplan–Meier method with median survival times and 95% confidence intervals (CIs). Associations between molecular subtype and selected clinicopathological parameters (grade, axillary lymph node status, BRCA status, family history) were evaluated using Fisher’s exact test. Bonferroni correction was applied for multiple pairwise comparisons.

All statistical analyses were performed in the statistical program R (version 4.3.1 (16 June 2023 ucrt)—“Beagle Scouts”; Copyright (C) 2023 the R Foundation for Statistical Computing; platform: x86_64-w64-mingw32/x64 (64-bit)). (available at www.r-project.org (accessed on 21 August 2023)).

Subgroup analyses involving molecular subtypes were considered exploratory, given the small sample sizes in several categories, and results were interpreted with caution.

## 3. Results

### 3.1. Patient Demographics

A total of 191 male breast cancer patients were included. Age data were available for 185 men, with a mean age of 65.17 ± 10.96 years and a median of 66 years (range 29–90). The majority were ≥50 years (91.6%), while 8.4% were younger than 50. A positive family history of breast cancer was recorded in 11 patients (5.8%), whereas 174 (91.1%) had no documented family history (Table 1).

### 3.2. Tumor Stage and Nodal Status

Regarding primary tumor size (pT), T2 tumors were most frequent (36.1%, 69/191), followed by T4 tumors (26.2%) and T1 subcategories (29.3%). Most patients presented without distant metastasis (M0, 96.3%, 184/191), while positive axillary lymph nodes were found in 51.3% (98/191) of patients (Table 2).

The mean number of removed axillary lymph nodes was 13.85 (range 0–33), with a mean of 2.43 positive nodes (range 0–25).

### 3.3. Histologic Grade and Receptor Status

**Figure 1:**
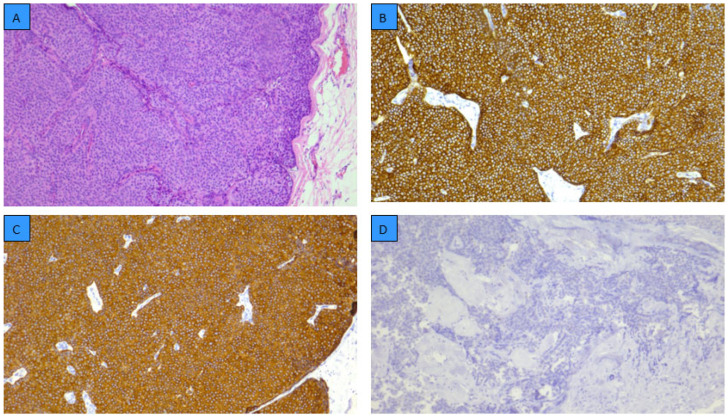
Representative histopathological and immunohistochemical features of invasive solid papillary carcinoma of the male breast. (**A**) Hematoxylin and eosin staining showing invasive solid papillary carcinoma, grade 2. (**B**) E-cadherin positivity confirms epithelial differentiation. (**C**) CK8/18 positivity. (**D**) p63 negativity supporting invasive growth. Original magnification ×100.

Most tumors were Grade 2 (77.5%, 148/191), while Grade 1 and Grade 3 represented 9.4% and 6.3%, respectively. Representative histopathological features and immunohistochemical profiles of male breast cancer are presented in Figure 1.

Hormone receptor positivity was high: ER-positive in 72.3% (138/191) and PR-positive in 88.0% (168/191). HER2 overexpression was observed in 11.0% (21/191). A high Ki-67 index (≥15%) was documented in 28.8% (55/191). These data are summarized in Table 3.

While hormone receptor positivity was frequent, a subset of tumors could not be incorporated into luminal subtype categories due to missing HER2 and/or Ki-67 data.

### 3.4. Molecular Subtypes

Based on immunohistochemical surrogate markers, tumors were classified as luminal A-like (7.3%, 14/191), luminal B-like (23.0%, 44/191), HER2-positive (11.0%, 21/191), triple-negative (2.6%, 5/191), luminal NOS (52.9%, 101/191), and missing subtype 31% (6/191), as shown in Table 4.

Although hormone receptor positivity was observed in the majority of tumors, a large proportion of cases were assigned to the luminal NOS category due to incomplete Ki-67 and/or HER2 data, particularly among patients diagnosed in earlier years of the study period. Consequently, these tumors could not be reliably subclassified into luminal A-like or luminal B-like groups.

As luminal subtype classification required complete biomarker data, the proportion of tumors classified as luminal A-like, luminal B-like, or luminal NOS was lower than the overall rate of hormone receptor positivity.

#### 3.4.1. Subtype and Nodal Status

Rates of axillary lymph-node positivity ranged from 35.7%, 5/14 (luminal A-like) to 61.4%, 62/101 (luminal NOS). The association between subtype and nodal status was not statistically significant (Fisher’s exact test, *p* = 0.144).

#### 3.4.2. Subtype and Histologic Grade

A significant association was observed between molecular subtype and grade (Fisher’s exact test, *p* = 0.045). Higher-grade tumors were more common in HER2-positive and triple-negative subtypes, whereas luminal A-like and luminal B-like tumors were predominantly Grade 2. Pairwise comparisons suggested that differences between luminal B-like and triple-negative groups approached statistical significance after correction, in line with the more aggressive biology of non-luminal subtypes.

#### 3.4.3. Subtype and BRCA Status

Analysis of BRCA status by molecular subtype was limited to the 37 patients who underwent germline testing. The BRCA-wild-type group (BRCA = 4) predominated across luminal subtypes, particularly luminal B-like and luminal NOS tumors. Given the small number of mutation carriers and the limited size of individual subgroups, these findings should be interpreted as exploratory.

### 3.5. Treatment Characteristics

**Figure 2:**
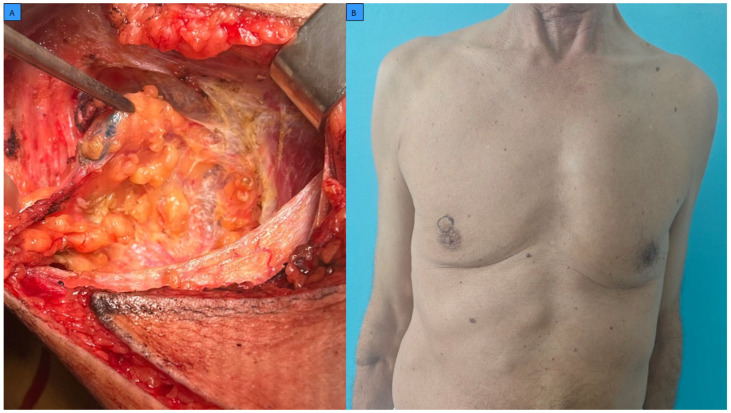
Representative surgical management of male breast cancer. (**A**) Intraoperative identification of the sentinel lymph node. (**B**) Preoperative photograph showing skin marking of tumor localization in a male breast cancer patient.

Surgery was performed in 185 patients (96.9%), most commonly mastectomy (78.5%). Representative preoperative and intraoperative aspects of surgical management in male breast cancer patients are shown in Figure 2.

Neoadjuvant treatment included chemotherapy (11.5%, 22/191), radiotherapy (8.4%), and hormonal therapy (1.6%). Adjuvant treatment consisted of chemotherapy in 36.1%, radiotherapy in 58.6%, and hormonal therapy in 68.1% (130/191). Hormonal therapy predominated, consistent with the high rate of hormone-receptor-positive disease.

Systemic therapy administered beyond the adjuvant setting was required in a minority of patients and generally reflected treatment for advanced or relapsed disease.

Among patients with HER2-positive breast cancer, a subset received HER2-targeted therapy, predominantly trastuzumab, reflecting treatment availability in the later years of the study period. The majority of HER2-positive cases diagnosed in earlier decades did not receive targeted therapy due to the lack of approved anti-HER2 agents at that time.

None of the patients with triple-negative breast cancer received immune checkpoint inhibitor therapy, as these treatments were not approved or routinely implemented for male breast cancer during the study period.

Although hormone-receptor-positive disease predominated, endocrine therapy was not administered to all eligible patients. This reflects the long retrospective study period, during which treatment recommendations evolved, as well as patient-related factors including advanced age, comorbidities, contraindications, treatment refusal, early postoperative death, and incomplete treatment documentation, particularly in earlier decades of the cohort.

### 3.6. Recurrence and Survival

Recurrence occurred in 58 patients (30.4%). The median follow-up was 53 months (range, 1–265). At last contact, 66.0% of patients were alive.

#### 3.6.1. Disease-Free Survival (DFS)

**Figure 3:**
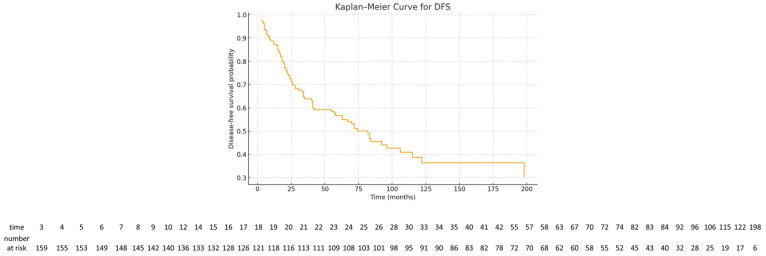
Kaplan–Meier disease-free survival curve. The number of patients at risk at selected time points is shown below the curve.

DFS analysis included 166 patients. Median DFS was 82 months (95% CI 57–115). DFS rates at 1, 2, 5, and 10 years were 87.3%, 72.5%, 56.7%, and 38.7%, respectively (Figure 3). The number of patients at risk was displayed below the Kaplan–Meier survival curves.

#### 3.6.2. Overall Survival (OS)

**Figure 4:**
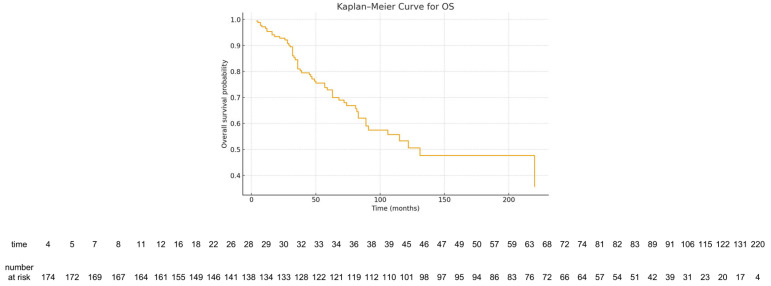
Kaplan–Meier overall survival curve. The number of patients at risk at selected time points is shown below the curve.

OS was analyzed for 184 patients. Median OS was 131 months. OS rates at 1, 2, 5, and 10 years were 95.3%, 92.8%, 72.9%, and 53.3%, respectively (Figure 4). The number of patients at risk was displayed below the Kaplan–Meier survival curves.

### 3.7. Genetic Findings

Germline genetic testing was performed in 37 of 191 patients (19.4%). Among tested patients, 7 (18.9%) harbored pathogenic germline variants. These included BRCA2 mutations in 3 patients, BRCA1 mutations in 1 patient, CHEK2 pathogenic variants in 2 of the 37 tested patients (5.4%), and a PALB2 pathogenic variant in 1 of the 37 tested patients (2.7%).

The remaining 30 tested patients (81.1%) had no pathogenic BRCA1 or BRCA2 variants detected and were classified as BRCA-wild type. This group corresponded to the predefined BRCA = 4 category, representing patients with negative germline BRCA testing. For analytical purposes, patients without available germline testing were classified as BRCA = 4, representing a presumed BRCA-wild-type group (80.6%, 154/191). This categorization was used to harmonize genetic analyses across the long study period.

## 4. Discussion

This large single-center series of 191 MBC patients provides a comprehensive overview of demographic, clinicopathological, genetic, treatment, and survival characteristics spanning over three decades. Our findings are broadly consistent with previous institutional and multicenter reports, while also offering region-specific insights relevant to understanding MBC in Southeastern Europe [1,2,3,5,12].

### 4.1. Age and Stage at Presentation

The mean age of 65 years and clear predominance of men ≥50 years correspond closely to international data, which place the typical age at diagnosis in the sixth or seventh decade [1,2,5,9]. More than half of the patients had axillary node involvement, and a substantial proportion presented with T2 or T4 tumors. This reflects well-documented diagnostic delays in men, driven by limited public awareness, minimal screening opportunities, and persistent misconceptions that breast cancer is a “female-only” disease [1,2,3]. Our results further underscore the need for educational initiatives that target both the general population and healthcare professionals.

### 4.2. Tumor Biology and Molecular Subtypes

Consistent with previous studies, our cohort showed very high estrogen and progesterone receptor positivity and a relatively low proportion of HER2-positive tumors. This confirms that MBC is largely an endocrine-responsive disease with a predominantly luminal biology [1,2,9,12,13].

The lower proportion of patients receiving endocrine therapy and the difference between overall hormone receptor positivity and luminal subtype assignment highlight the real-world limitations of long-term retrospective cohorts and should not be interpreted as biological inconsistency.

Molecular subtype distribution showed that luminal A/B-like tumors represented about one-third of cases with complete data, while more than half fell into the luminal-NOS category, reflecting limitations in available biomarker data inherent to this long-term retrospective cohort. HER2-positive and triple-negative tumors were uncommon, aligning with prior findings that triple-negative MBC is significantly rarer than its female counterpart [2,12].

It should be emphasized that the designation “luminal NOS” in this study reflects limitations in available immunohistochemical data rather than a distinct biological subtype and is conceptually unrelated to the histopathological term invasive ductal carcinoma, NOS.

Importantly, we observed a significant association between subtype and histologic grade, with high-grade tumors clustering within HER2-positive and triple-negative groups. This pattern is well established in the literature and supports the biological differences between luminal and non-luminal MBC [12,13]. Although the association between subtype and nodal positivity was not statistically significant, trends suggested biologically plausible differences that may merit further investigation in larger datasets.

### 4.3. Hereditary Component and Genetic Findings

Only 5.8% of patients reported a family history of breast cancer, a figure similar to other series where 5–15% of MBC is attributed to hereditary predisposition [6,8,10]. In our cohort, most tested patients belonged to the BRCA-wild-type group, but approximately one-fifth showed non-wild-type or indeterminate results, and additional germline pathogenic variants were detected in CHEK2 and PALB2.

Although gene-level categorization was limited, this distribution is consistent with the existing literature, indicating that BRCA2 represents the predominant hereditary driver of MBC, followed by smaller contributions from BRCA1, CHEK2, and PALB2 [5,11]. Moreover, our subtype-stratified analysis demonstrated that the BRCA-wild-type group predominated in luminal NOS and luminal B-like subtypes, whereas non-wild-type findings were more heterogeneously distributed across other categories—a pattern also reported in previous retrospective genetic cohorts. Together, these findings underscore the ongoing importance of recommending genetic counseling for all men diagnosed with breast cancer. Several large genetic and guideline-driven studies further emphasize that universal referral for genetic counseling should be considered, irrespective of family history, given the substantial prevalence of clinically actionable germline mutations identified even in apparently sporadic cases [4,16,17].

### 4.4. Treatment Patterns and Survival

Treatment patterns in our cohort align closely with contemporary recommendations. Surgery was performed in the vast majority of patients, typically a mastectomy, which remains standard practice due to small breast volume and frequent advanced presentation in men. Adjuvant hormonal therapy was widely used and is supported by American Society of Clinical Oncology (ASCO) guidelines advocating tamoxifen as first-line therapy for hormone-receptor-positive MBC [3,4].

Chemotherapy and radiotherapy were administered according to stage and risk features, mirroring treatment principles used in female breast cancer. Systemic therapy beyond the adjuvant setting was required only in a minority of patients, reflecting treatment for metastatic or recurrent disease.

Survival outcomes showed a median DFS of 82 months and a median OS of 131 months. Despite relatively favorable long-term OS, the steady decline in DFS curves, particularly beyond 5–10 years, underscores that MBC carries a meaningful risk of late recurrence—consistent with prior long-term follow-up studies demonstrating that hormone-receptor-positive male breast cancer is associated with a persistent risk of late recurrence and mortality extending beyond 15–20 years after diagnosis [18,19]. These findings support ongoing discussions regarding extended endocrine therapy in selected high-risk luminal cases.

### 4.5. Comparison with Contemporary Literature

When compared with recent series from Europe, North America, and Asia, our results demonstrate a high degree of concordance, particularly regarding age distribution, luminal tumor biology, and the prevalence of nodal involvement at diagnosis [1,2,5,12,13].

Recent European retrospective studies provide an important comparative framework. An Italian 18-year single-institution experience reported by Taraschi et al. confirmed a predominance of luminal tumors and mastectomy as the most common surgical approach, similar to our findings, although with a slightly lower proportion of locally advanced disease at diagnosis [20].

A two-center French analysis by Charpentier et al. also confirmed the predominance of luminal subtypes in male breast cancer but reported a lower proportion of nodal involvement, which may reflect differences in healthcare access, diagnostic pathways, or referral patterns between Western European and Southeastern European populations [21].

A large multicenter Spanish cohort spanning 27 years, reported by Dobato Portoles et al., demonstrated clinicopathological characteristics closely aligned with our cohort, including advanced age at diagnosis and frequent nodal positivity, reinforcing the notion that delayed diagnosis in men remains a widespread challenge across different healthcare systems [22].

Furthermore, a recent single-institution clinicopathological study from Asia by DE Sarkar et al. highlighted potential population-specific differences in tumor biology, genetic testing practices, and treatment strategies, underscoring the influence of regional and methodological factors on reported outcomes [23].

Emerging evidence emphasizes that although MBC shares many features with postmenopausal female breast cancer, it exhibits unique patterns in genetics, psychosocial burden, and survivorship needs [1,2,10]. Our data reinforce this concept, especially the relatively high frequency of locally advanced disease and the notable proportion of luminal NOS tumors, which may reflect regional practice patterns, diagnostic challenges, or incomplete biomarker reporting. Psychosocial burden and survivorship challenges remain underreported in men with breast cancer and appear to differ substantially from those observed in female breast cancer populations, highlighting the need for sex-specific survivorship research and supportive care strategies [10,24,25].

### 4.6. Limitations

This study has inherent limitations due to its retrospective design, including missing biomarker data, incomplete details on genetic results, and a lack of information on comorbidities and cause-specific mortality. Molecular subtypes were assigned using immunohistochemical surrogates rather than intrinsic molecular profiling, which may limit direct comparability with studies based on genomic classification. Subgroup analyses based on molecular subtype and genetic status were limited by small sample sizes, particularly in non-luminal categories. Accordingly, inferential statistics were applied in an exploratory manner, and observed associations should be interpreted with caution. Larger, multicenter studies are required to validate these findings. Nevertheless, the large consecutive cohort and extended follow-up period provide robust real-world evidence that contributes to the growing understanding of male breast cancer.

## 5. Conclusions

In this retrospective cohort of 191 male breast cancer patients, most men were diagnosed at an older age and frequently presented with nodal involvement and intermediate-grade tumors. The tumor profile was strongly dominated by hormone-receptor-positive, luminal-type disease, whereas HER2-positive and triple-negative subtypes were uncommon. Disease recurrence occurred in roughly one third of patients, and long-term survival—although encouraging—shows room for improvement, particularly given the substantial proportion presenting with locally advanced disease.

These findings underscore the importance of increasing public and professional awareness to facilitate earlier diagnosis in men, ensuring routine referral for genetic counseling, and applying subtype-directed, guideline-concordant treatment strategies. Together, these measures have the potential to improve outcomes and refine future approaches to male breast cancer management.

## Figures and Tables

**Table 1:** Demographic characteristics.

Variable	n/N (%) or Mean ± SD (Range)
Age (years), mean ± SD (range)	65.17 ± 10.96 (29–90)
Age ≥ 50 years	175/191 (91.6%)
Family history of breast cancer (yes)	11/191 (5.8%)

**Table 2:** Primary tumor stage and nodal characteristics. *ALN—axillary lymph node.*

Variable	Category	n (%)
p(T)	T2	69 (36.1)
	T1 subcategories (1, 1.2, 1.3)	56 (29.3)
	T4.2	50 (26.2)
p(N)	N0	78 (40.8)
	N1	83 (43.5)
	N2–3	24 (12.6)
p(M)	M0	184 (96.3)
	M1	1 (0.5)
ALN	Positive	98 (51.3)
	Negative	87 (45.6)

**Table 3:** Histologic grade and biomarker expression.

Variable	Category	n (%)
Grade	1	18 (9.4)
	2	148 (77.5)
	3	12 (6.3)
ER.	Positive	138 (72.3)
PR.	Positive	168 (88.0)
HER2	Positive	21 (11.0)
Ki67	High (1)	55 (28.8)

**Table 4:** Molecular subtype distribution.

Subtype	n (%)
Luminal A-like	14 (7.3)
Luminal B-like	44 (23.0)
HER2-positive	21 (11.0)
Triple-negative	5 (2.6)
Luminal NOS	101 (52.9)
Missing	6 (3.1)

## Data Availability

Data supporting the findings of this study are available from the corresponding author on reasonable request. The dataset is not publicly available due to patient privacy and ethical restrictions.

## Abbreviations

The following abbreviations are used in this manuscript:

MBC	Male breast cancer
ER	Estrogen receptor
PR	Progesterone receptor
HER2	Human EPIDERMAL Growth Factor Receptor 2
DFS	Disease-free survival
OS	Overall survival
CI	Confidence Interval
TNM	Tumor–Node–Metastasis Classification
ASCO	American Society of Clinical Oncology
ALN	Axillary Lymph Nodes
NOS	Not otherwise specified
WHO	World Health Organisation
TNBC	Triple-Negative Breast Cancer
